# The Royal Military Benevolent Fund

**Published:** 1900-08-04

**Authors:** 


### THE ROYAL MILITARY BENEVOLENT FUND.

It is officially announced that the Queen, the Prince and Princess of Wales, and the Duke and Duchess of York have withdrawn their patronage from the Royal Military Benevolent Fund, 193, Queen's Gate, S.W.

The following particulars, preceded by an asterisk, appear in Burdett's "Hospitals and Charities, 1899." The asterisk indicated that no return had been received for the current year, although repeated applications for information had been made. The particulars given are:—

"*Royal Military Benevolent Fund.—Founded 1875; school opened 1890. Patrons: H.M. the Queen and H.R.H. the Prince of Wales. Founder and treasurer: Mrs. Ellis-Williams, 193, Queen's Gate, S.W. Provides annuities for widows and unmarried orphan daughters, and education and maintenance for daughters of officers in the Army and Royal Marines; 86 annuitants; 40 pupils in the school. Admission: Pupils by selection by committee, and payment of £12 12s. a year. Annuitants by election by subscribers. Income (1896-7): £2,360. Expenditure: No return."

A committee, of which General Laurie, M.P., is chairman, and Mr. William Plender hon. secretary, have, we understand, been engaged for some time past in investigating the affairs of the Royal Military Benevolent Fund.

